# Supplementary material for: The OpenPicoAmp: An Open-Source Planar Lipid Bilayer Amplifier for Hands-On Learning of Neuroscience
Source: PLoS One. 2014 Sep 24;9(9):e108097. doi: 10.1371/journal.pone.0108097 (PMC4176719; doi:10.1371/journal.pone.0108097)
Supplement: File S3 — Document detailing the practical implementation of the amplifier and a ready to print layout for the printed-circuit board (scale 1∶1). (PDF) [file pone.0108097.s003.pdf]

Implementation of the amplifier

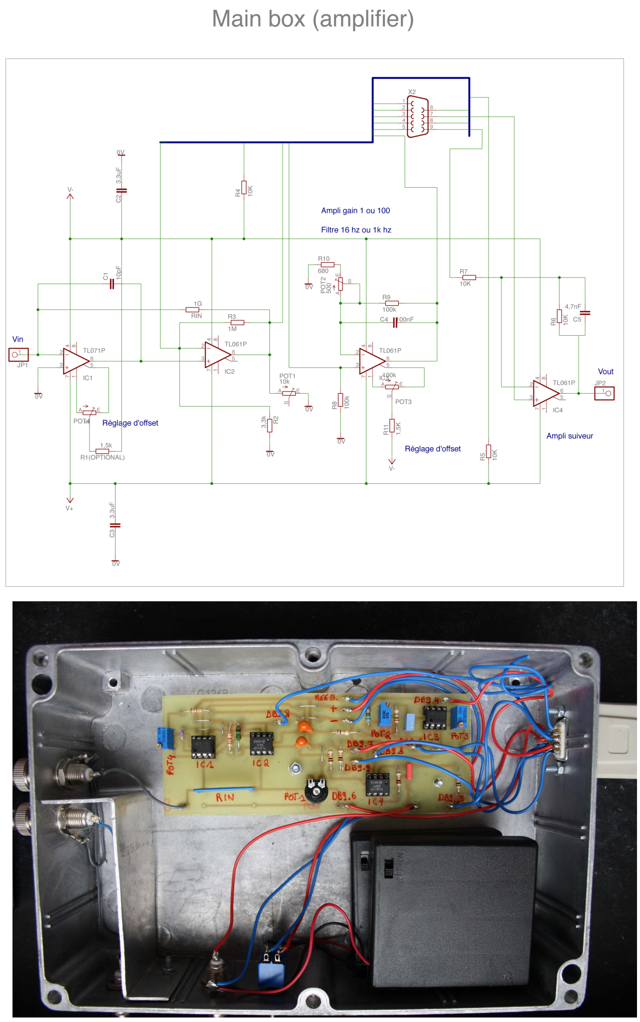

Figure 7: Circuit schematics and implementation of the amplifier in the main box. The blue line represents a bus. The wiring can be followed by opening the schematic file (S2_OpenPicoAmp.sch) and using the "show" tool in the Eagle software.

The OpenPicoAmp can be implemented by following these steps :

- order or print the board circuit (PCB) according the proposed layout (given at end of this document).
- individual components have to soldered to the PCB according to the board layout contained in the file S1 (S1_OpenPicoAmpBoard.brd). Respect carefully the polarity of the capacitors C2 and C3.
- the main box needs to be drilled to install the following : two BNC connectors to the recording chamber, one BNC connector for the input signal, one BNC connector for the output signal, a two positions power switch and a DB9 connector for the remote control.

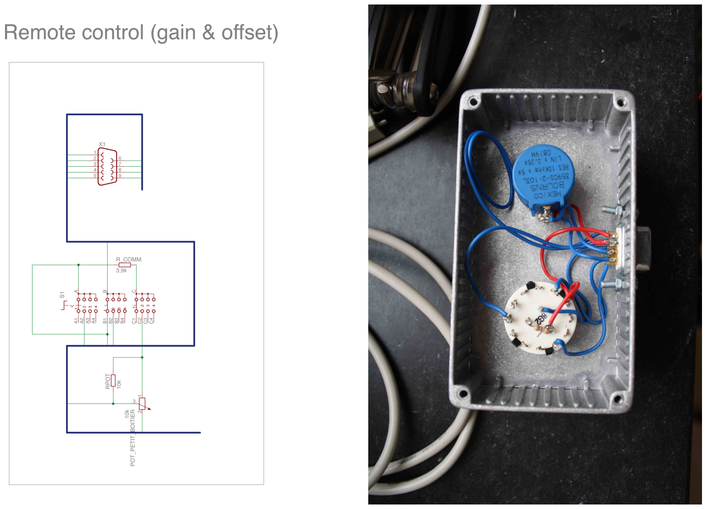

Figure 8: Circuit schematics and implementation of the remote control. The blue line represents a bus. The wiring can be followed by opening the schematic file (S2_OpenPicoAmp.sch) and and using the "show" tool in the Eagle software.

- the PCB board should be installed in the enclosure of the main box (fig. 7) and connected to the other components, including the two 6V battery holders.
- the next step is to make the remote control, which contains a 10 $k\Omega$ potentiometer, a 3 poles 4 positions rotary switch, a 10 $k\Omega$ resistor, a 3.9 $k\Omega$ resistor and a DB9 connector. The corresponding schematics and the proposed implementation is shown on fig. 8.
- the recording chamber needs to be drilled to install the two BNC connectors.
- connect the remote control to the amplifier with a RS232 DB9 cable and connect the main box to the recording chamber using the two BNC-BNC adapters (fig. 9).
- switch the amplifier on.
- on the first use, the OpenPicoAmp needs to be calibrated by adjusting the potentiometers POT1, POT2, POT3 and POT4. For that a 50 Ω BNC terminator should be connected to the input of the amplifier, the model circuit (composed of a 1 $G\Omega$ resistance with a 220 pF capacitor in parallel) should be connected to the BNC in the recording chamber (lid closed) and the output of the amplifier should be connected to an oscilloscope.
- the potentiometer of the remote control should be precisely halfway, with 0V at its output.
- with the amplifier set on gain 1 mV/pA, use POT4 to adjust the output at 0V.
- with the amplifier set on gain 100 mV/pA, use POT3 to adjust the output at 0V.
- remove the 50 Ω BNC terminator and connect the input of the amplifier to an ajustable DC source.
- with the amplifier set on gain 100 mV/pA and a constant voltage of 10 mV at the input of the amplifier, use POT2 to adjust the output at 1 V.
- connect the input of the amplifier to a function generator delivering a 500 Hz 10 mV peak to peak triangular signal.
- with the amplifier set on gain 1 mV/pA, use POT1 to adjust the shape of the signal so it appears as square as possible.

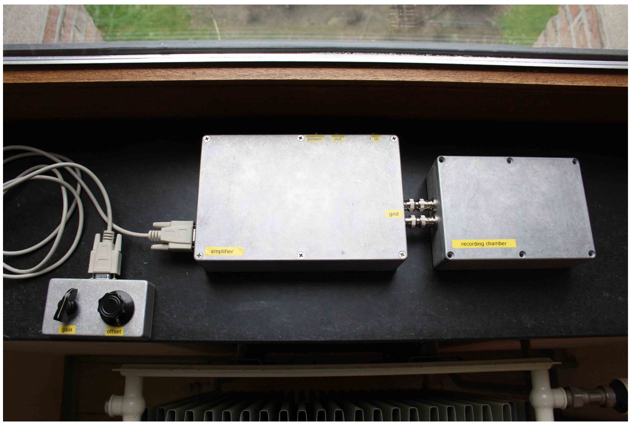

Figure 9: The complete setup including the amplifier, the recording chamber and the remote control.

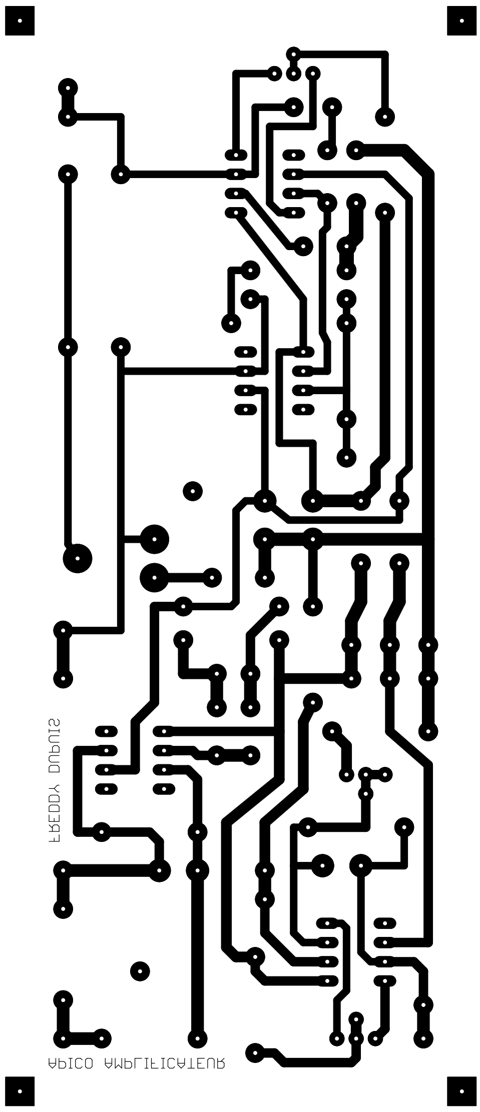
FREDDY DUPUIS
APICO AMPLIFICATEUR
