# Supplementary figures and images for: The OpenPicoAmp: An Open-Source Planar Lipid Bilayer Amplifier for Hands-On Learning of Neuroscience
Source: PLoS One. 2014 Sep 24;9(9):e108097. doi: 10.1371/journal.pone.0108097 (PMC4176719; doi:10.1371/journal.pone.0108097)

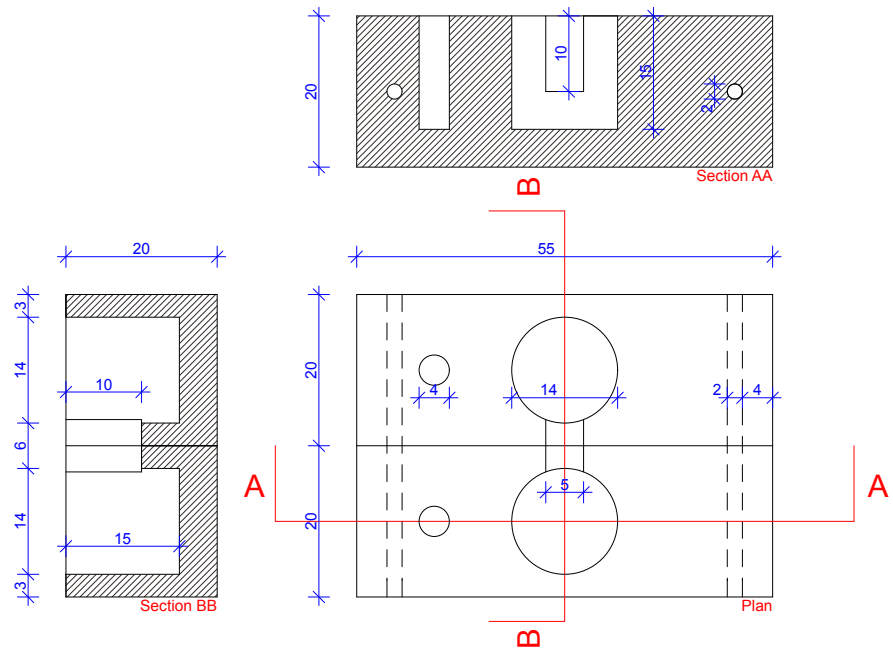

Supplement: File S5 — Document detailing the design of our bilayer chamber (all dimensions given in mm ) and a STL file allowing the 3D printing of the parts. (GZ) [file pone.0108097.s005.gz › S4_BLMchamber/S4_BLMchamberBlueprint.pdf]
