# Supplementary material for: The OpenPicoAmp: An Open-Source Planar Lipid Bilayer Amplifier for Hands-On Learning of Neuroscience
Source: PLoS One. 2014 Sep 24;9(9):e108097. doi: 10.1371/journal.pone.0108097 (PMC4176719; doi:10.1371/journal.pone.0108097)
Supplement: File S6 — Document describing the preparation of the Teflon film, the assembly of the bilayer chamber and the making of the electrodes. (PDF) [file pone.0108097.s006.pdf]

Preparation of Teflon films with aperture.

Assembly and cleaning of the bilayer chamber.

Fabrication of Ag/AgCl electrodes and agar bridges.

Preparation of a piercing tool.

For this purpose we modified a point of a 27-gauge syringe needle to a conical shape using consequent polishing using P240, P600 and finally P2500 sandpaper. Under microscope, the opening of the point should be plane.

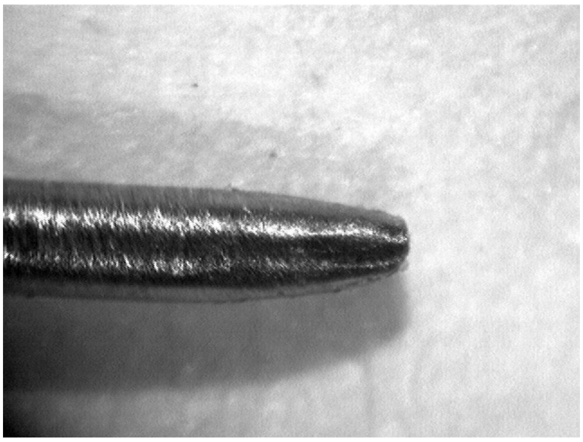

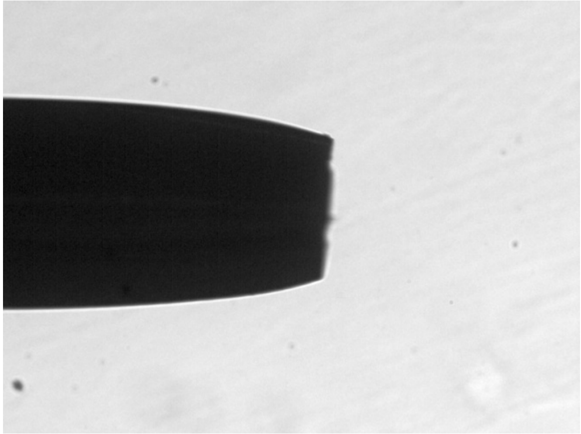

Making a hole

A sheet of Teflon film is placed on a block of hard but ductile material (plumb, delrin or wood). Piercing tool is then positioned against the film vertically and small punch is applied to the back of the needle. The channel of this piercing tool should be cleared of Teflon after each punch using mandrel.

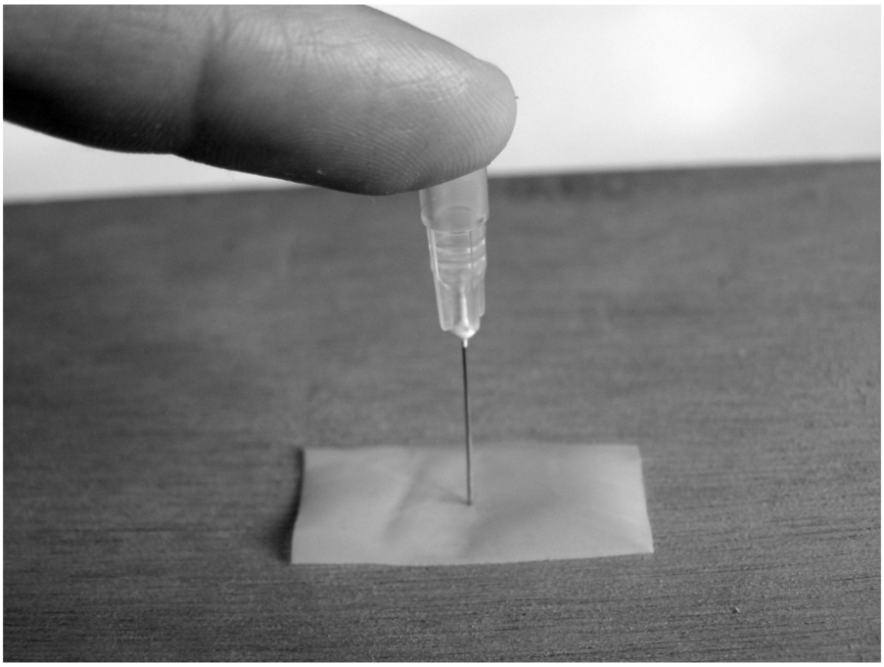

Such a piercing tool makes reproducible apertures of 100-150 µm in diameter. Before use, the apertures are polished using a human hair and then the films are cleaned with detergent and are rinsed in de-ionized water. One can note that polishing of the hole makes it slightly larger.

<u>Not polished</u>

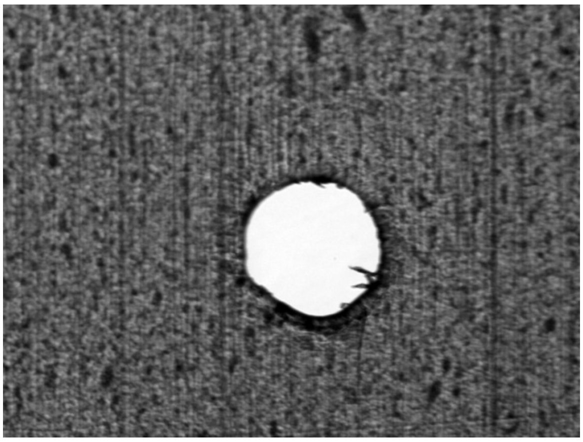

<u>Polished with a hair</u>

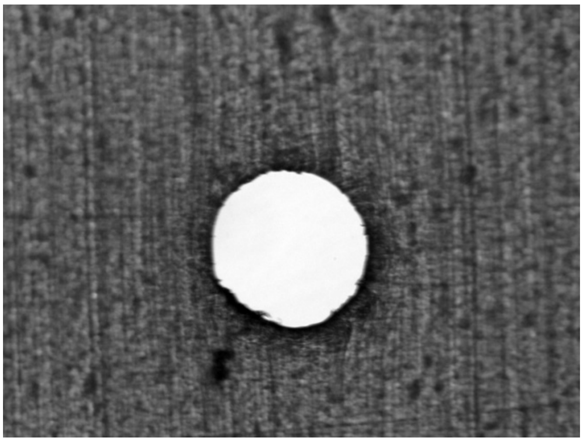

Sometimes piercing tool makes uneven apertures (especially if pierced Teflon remains if the channel of the needle), and such Teflon films should not be used:

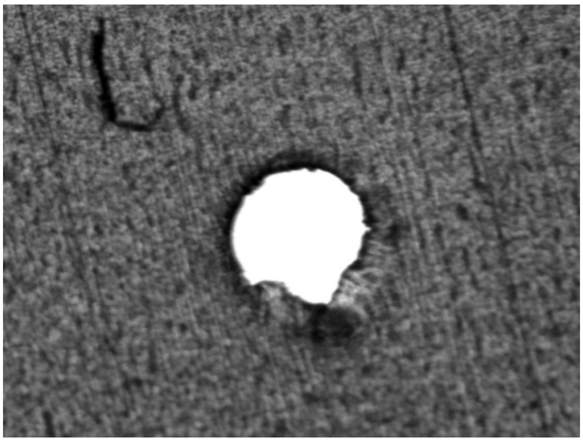

Chamber assembly

Both halves of the chamber are smeared with silicone grease as shown in the figure:

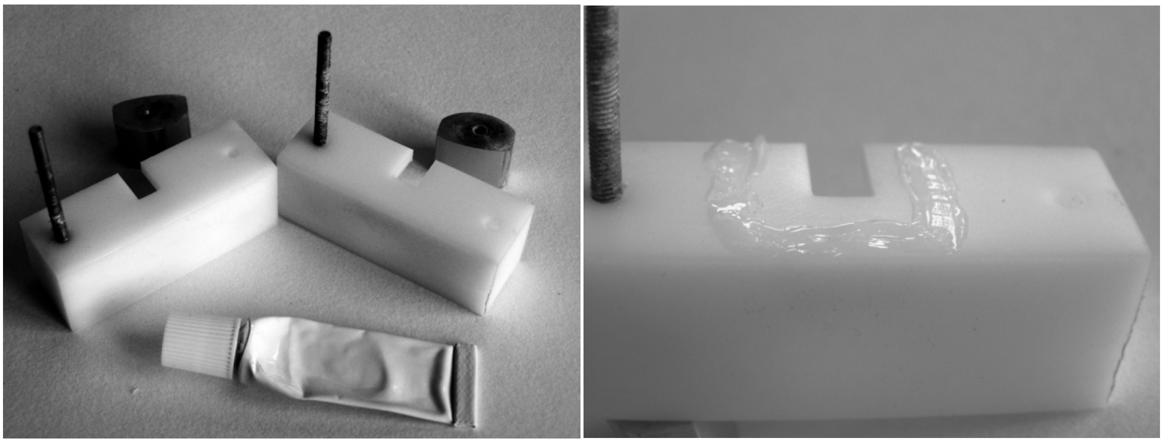

Then the film with an aperture is placed between the halves of the chamber and fixed with the screws. The upper edge of the film should be 3-5 mm above the level of the chamber:

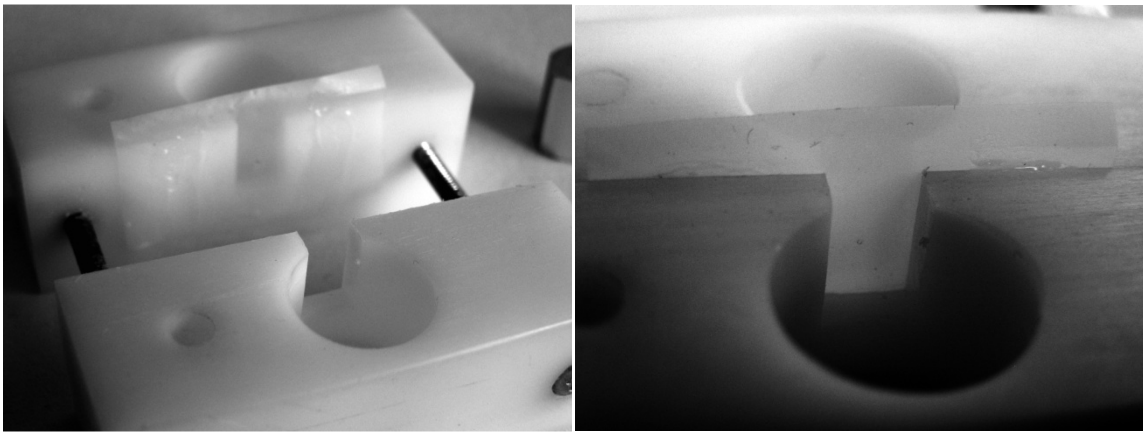

In our experience, one or two films (and accordingly chambers) usually get destroyed during any particular session due to inaccurate students' handling. Accordingly, we keep a stock of pierced films that permits immediate reconstitution of broken chambers. For this reason, the use of grease is essential; it assures good insulation while it does not introduce additional adhesion forces making disassembly of the chamber easy.

Cleaning

The chambers used in this laboratory are cleaned with a liquid detergent. Attention should be made to force the detergent to go through the aperture. Chambers are rinsed thoroughly under running tap water then in deionized water and dried before use.

Fabrication of Ag/AgCl electrodes

A 1 mm electrode pin is soldered to a 7-cm-long Ag wire of 0.5 mm in diameter (catalog no. 327026, Sigma-Aldrich). Four centimeters of silver wire are folded into a helix (on a match, for example), cleaned in alcohol, and then covered with AgCl by putting the electrodes for 1 h into any commercial bleach solution containing 10% available chlorine (e.g. Clorox, eau de Javel, or equivalent). To minimize potential offsets, electrodes should be chloridized in pairs with their pins interconnected.

Fabrication of agar bridges

Polyethylene tubing with approximately 1 mm inner diameter (Catalog no. 14-170-11C, Fischer Scientific) is cut into 4 cm long pieces which then are folded into arcs by brief heating the center of every piece on a Bunsen burner. Agarose (catalog no. A9539, Sigma-Aldrich) is melted in 1 M KCl on a hotplate. While the agarose is still hot, polyethylene arcs are fully filled with agarose and then immediately placed into ice-cold 1 M KCl for storage.
