# Supplementary material for: The OpenPicoAmp: An Open-Source Planar Lipid Bilayer Amplifier for Hands-On Learning of Neuroscience
Source: PLoS One. 2014 Sep 24;9(9):e108097. doi: 10.1371/journal.pone.0108097 (PMC4176719; doi:10.1371/journal.pone.0108097)
Supplement: File S8 — The student hand-out for the experiments. (PDF) [file pone.0108097.s008.pdf]

Lab session III : Ionic channels

3.1 Introduction

The existence of an electrical membrane potential relies on two properties of the cell membrane :

- the selective ionic permeability due to the existence of ionic channels.
- existence of transmembrane concentrations gradients for the permeant ions.

The aim of this session is to study the passive properties of an artificial cell membrane and to allow the direct observation of the activity of single ionic channels in different ionic conditions.

3.1.1 Prerequisites

The reading of the course notes on the electrical equivalent circuit of the cell membrane is a prerequisite for this lab session. In addition, a short video is available to introduce the different devices you'll have to use during the experiment.

3.1.2 Goals

- Study the electrical response of a RC circuit when it is submitted to constant voltage or a triangular signal.
- Study the electrical response of a planar lipid bilayer when it is submitted to constant voltage or a triangular signal.
- Observe the electrical currents produced by the openings of a single ionic channel.
- Study the ohmic behavior of an ionic channels in different ionic conditions.

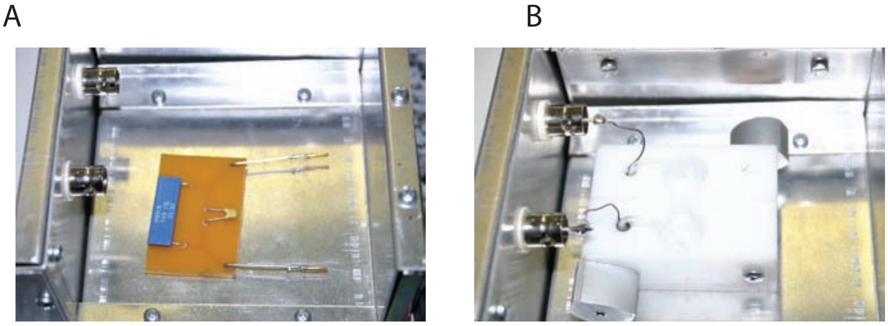

Figure 3.10: (A) Test circuit used for the calibration of the amplifier, it is composed of a 1 $G\Omega$ resistor with a 220 pF capacitor in parallel. The circuit board has two connectors that can be inserted in the BNC connectors of the amplifier. (B) The bilayer chamber which is composed of two compartments *cis* and *trans*. Each compartment is connected to the amplifier though an Ag-AgCl electrode and a salt bridge. A Teflon film is sealed with silicon grease between the two blocks, a circular 200 μm aperture is visible at the centre of the film separating the two compartments.

3.2 Material

The following apparatus will be used in this lab sessions :

- an **amplifier**, acting as a current to voltage converter, allowing the measure of the current flowing through the lipid bilayer at a given potential. Two modes of operation are available through a two position switch on the remoter control : one with a 1 mV/pA gain combined with a 1 kHz low-pass filter and the other with a 100 mV/pA gain combined with a 16 Hz low-pass filter. In addition, the amplifier offset can be adjusted using the rotating knob on the remote control.

- a **test circuit** composed of a 1 $G\Omega$ resistor with a 220 pF capacitor in parallel (fig. 3.10.A)

- the **bilayer chamber** which is composed of two compartments milled in two blocks of polyoxymethylene which are pressed together by a pair of screws. Each compartment is connected to the amplifier though

an Ag-AgCl electrode and a salt bridge. A Teflon film is sealed with silicon grease between the two blocks. Before insertion of the film in the chamber, a circular 200 μm aperture has been first etched in the film (visible at the centre of the film separating the two compartments). (fig. 3.10.B).

- a two channel digital **oscilloscope** (preset for the firsr measurement: time base 1 ms/DIV, CH1 : 2 mV/DIV, CH2 : 2 V/DIV, DC mode on both channels).
- a **function generator** (preset to supply a 500 Hz and 10 mV peak to peak triangular signal)
- a switchable **DC source**, tunable with a rotating knob ($\Delta V = \pm 120 mV$, 12 mV/turn)
- a NI USB 6008 **analog to digital converter** allowing the digitization of the measured signal with a 12 bits precision in a -10 V à +10 V interval.
- two lab **pipettes** (1 ml and 10 μl)
- a stock solution of **lipids**, 3-sn-phosphatidylcholine diluted in n-octane (20 mg/ml)
- a 1 nM **gramicidin A** stock solution, diluted in ethanol. Gramicidin A dimers forms ionic channels in lipid bilayers, which are selective for monovalent cations. (fig. 3.12.A).
- 1 M and 0.1 M **NaCl** solutions.

3.3 Report

The following points should be clearly explained in the written report you have to submit at the end of the session:

- how does the RC circuit react when submitted to a 500 Hz 10 mV peak to peak triangular signal or continuous 120 mV voltage signal. Provide your detailled calculations for the test circuit (1 $G\Omega$ resistor with a 220 pF capacitor in parallel). Why is the output signal of the amplifier is a square signal ?

- provide the calculations allowing the estimation of the bilayer diameter, knowing the specific capacitance of a lipid bilayer (0.5 $\mu F/cm^2$) and the intensity of the capacitive current.
- provide the calculations allowing the estimation of the bilayer resistivity, knowing that the typical thickness of a planar lipid bilayer is around 10 nm .
- explain quantitatively the observed change in the reversal potential of the unitary current observed in asymmetric ionic conditions.

3.4 Measurements

3.4.1 Calibration

- connect the RC circuit to the amplifier with the gain at 1 mV/pA (fig 3.10.A).
- using the oscilloscope, check that the input signal is a 500 Hz and 10 mV peak to peak triangular signal.
- on the CH2 of the oscilloscope (1 V/div), observe the output signal of the amplifier which is proportional to the total current flowing through the circuit. Based on the observed current trace, provide an estimation the capacitance present in circuit.
- put the switch situated on the rear panel the DC source (blue box) on the high position. This replaces the signal coming from the function generator by a constant voltage.
- put the switch situated on the front panel of the DC source (blue box) on the middle position (0 mV) and use the offset knob on the remote control to adjust the output signal of the amplifier at 0 V.
- put the switch situated on the front panel of the DC source (blue box) on the high position. This replaces the signal coming from the function generator by a constant voltage (($\Delta V = \pm 120 mV$) which can be set with the rotating knob (12 $mV/turn$).
- on the oscilloscope (CH2 on 100 mV/div), observe the output signal which is proportional to the total current flowing through the circuit. Based on the observed current trace, provide an estimation the resistance present in circuit.

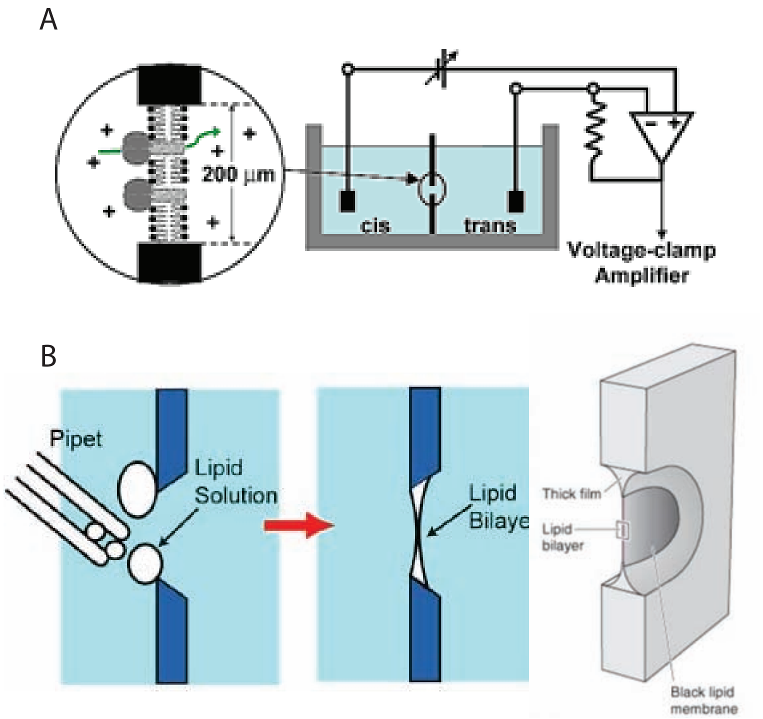

Figure 3.11: Experimental setup. (A) The two compartments of the experimental chamber are called *cis* and *trans*, the *trans* side is always defined as zero voltage (corresponding to the usual convention in electrophysiology where the reference potential is on the extracellular side). The voltage signal is applied on the *cis* side while the *trans* side is held at 0 V by the current to voltage converter acting as a virtual ground. (B) Lipids are injected over the aperture in the Teflon film separating the two compartments. The bilayer is formed by spontaneous thinning

3.4.2 Planar lipid bilayer

- fill both compartments with the 1 M NaCl solution and connect the bilayer chamber to the amplifier (fig 3.10.B) and set the amplifier at the 1 mV/pA gain.

- put a 500 Hz and 10 mV peak to peak triangular signal at input of the amplifier by putting the switch situated on the DC source on the low position (fig. 3.11.A).

- inject 2 μl of a 20 mg/ml solution of 3-sn-phosphatidylcholine diluted with n-octane over the aperture in the Teflon film. The bilayer is formed by spontaneous thinning (fig. 3.11.B). If the lipid bilayer does not form within a few minutes after adding the lipids, agitate the electrolyte solution to speed up the thinning process. This can be achieved by producing small air bubbles near the aperture with a 10 μl pipette. Monitor the bilayer formation by evaluating the capacitive current during the application of a triangular voltage signal (500 Hz, 10 mv peak to peak). As a the planar lipid bilayer is forming in the central part of the aperture, the capacitive current will increase and then stabilize (amplifier gain at 1 mV/pA and oscilloscope CH2 on 1 V/div). Based on the observed current, estimate the capacitance of the bilayer. A stable lipid bilayer formed inside the 200 μm aperture in the teflon film will typically produce a capacitance in the range of hundreds of pF. The capacitance of the bilayer is the first criteria for proceeding with an experiment. Low capacitance reflects a bilayer that has not completely formed or that is overly thick. In that case further agitation is needed to speed up the thinning process (fig. 3.11.B).

- knowing the specific capacitance of a lipid bilayer (0.5 $\mu F/cm^2$) and the intensity of the capacitive current, estimate the bilayer diameter (which should be slightly lower than the aperture diameter due to the presence of the annulus at the periphery of the bilayer).

- put the switch situated on the rear panel the DC source (blue box) on the high position. This replaces the signal coming from the function generator by a constant voltage. Set the amplifier at the 100 mV/pA gain.

- put the switch situated on the front panel of DC source (blue box) on the middle position (0 mV) and use the offset knob on the remote control to adjust the output signal of the amplifier at 0 V.

- put the switch situated on the front panel of the DC source (blue box) on the high position and apply 120 mV at the input of the amplifier. The final bilayer stability test is the amount of current flowing across the channel-free bilayer in response to test potentials in the voltage range that is to be used in the experiment. If test potentials generate substantial currents across the membrane in the absence of ionic channels, the bilayer is deemed unstable and should be broken and reformed before beginning an experiment. In our case, the total current at a constant 120 mV voltage should be below 2 pA (amplifier gain at 100 mV/pA and oscilloscope CH2 on 100 mV/div).

- knowing that the typical thickness of a planar lipid bilayer is around 10 nm, estimate of the bilayer resistivity and demonstrate that lipids act as an insulator.

3.4.3 Unitary currents

- break the previously obtained channel-free bilayer.

- add 1 μl of the 1 nM stock solution of gramicidin in each compartment (fig. 3.12.A).

- form the lipid bilayer ($R_m \approx 100G\Omega$, $C_m \approx 100pF$)

- observe the membrane current at a constant voltage of$\Delta\ V = \pm 120mV$, with the oscilloscope CH2 on 100 mV/div and 500 ms/div (amplifier gain at 100 mV/pA). You should be able to record unitary currents (fig. 3.12.B). The current mediated by each channel is proportional to the applied voltage; dividing the current flowing through each channel by the applied voltage gives the conductance of the channel which is around 15 pS in these experimental conditions.

- measure the unitary currents at different values of the holding potential to demonstrate their ohmic behavior.

- repeat the whole procedure in asymmetric ionic conditions (NaCl 1 M *cis* / 0.1 M *trans*). Estimate the reversal potential of the unitary currents in these experimental condition.

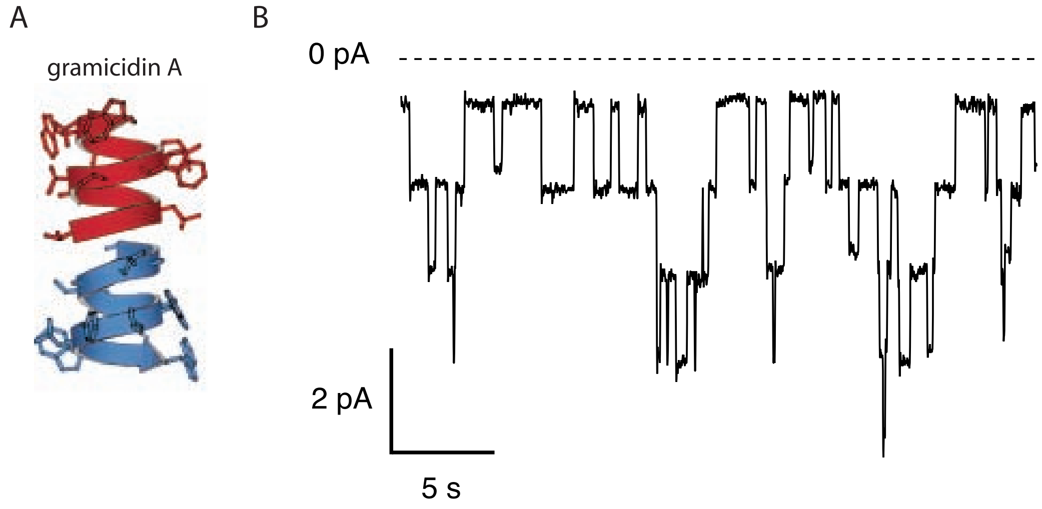

Figure 3.12: (A) Gramicidin A is a small peptide that forms ionic channels in lipid bilayers. When two gramicidin A molecules link transiently, they form an open ionic channel which is selective for cations. (B) After addition of gramicidin A, unitary currents can be recorded when the bilayer is submitted to constant voltage. In the presented recording, obtained at -120 mV, a maximum of four gramicidin A pores are open at the same time.
