# Supplementary material for: The OpenPicoAmp: An Open-Source Planar Lipid Bilayer Amplifier for Hands-On Learning of Neuroscience
Source: PLoS One. 2014 Sep 24;9(9):e108097. doi: 10.1371/journal.pone.0108097 (PMC4176719; doi:10.1371/journal.pone.0108097)
Supplement: File S9 — The student survey questions. The lecture material related to the laboratory session can be obtained upon request by contacting the corresponding author. (PDF) [file pone.0108097.s009.pdf]

Please fill **BEFORE** the lab session

Equivalent circuit of the passive cell membrane

	T	F
The intensity of the current flowing through the test circuit is higher when it is submitted to a the AC voltage compared to the DC voltage ?	☐	☐
The capacitive current is zero when the applied voltage is constant.	☐	☐
The total current is purely resistive when the applied voltage is constant.	☐	☐

Membrane capacitance of the planar lipid bilayer

	T	F
The electric field is constant inside the lipid bilayer and zero outside.	☐	☐
The capacitance of the bilayer is a function of its radius.	☐	☐
The observed capacitance decreases during the lipid bilayer formation.	☐	☐

Membrane resistance of the planar lipid bilayer

	T	F
Lipids are a good insulator.	☐	☐
The bilayer resistance changes in asymmetric ionic conditions compared to its value in symmetric ionic conditions.	☐	☐
Lipid resistivity is a function of the thickeness of the bilayer.	☐	☐

Ionic channels

	T	F
Ionic channels formed by gramicidin molecules behave as ohmic conductors.	☐	☐
The total conductance is obtained by multiplying the total number of channels by the unitary conductance and by the open propability.	☐	☐
Unitary currents are zero at a 0 mV holding potential in asymmetric ionic conditions.	☐	☐

Please fill **AFTER** the lab session

Equivalent circuit of the passive cell membrane

	T	F
The intensity of the current flowing through the test circuit is higher when it is submitted to a the AC voltage compared to the DC voltage ?	☐	☐
The capacitive current is zero when the applied voltage is constant.	☐	☐
The total current is purely resistive when the applied voltage is constant.	☐	☐

Membrane capacitance of the planar lipid bilayer

	T	F
The electric field is constant inside the lipid bilayer and zero outside.	☐	☐
The capacitance of the bilayer is a function of its radius.	☐	☐
The observed capacitance decreases during the lipid bilayer formation.	☐	☐

Membrane resistance of the planar lipid bilayer

	T	F
Lipids are a good insulator.	☐	☐
The bilayer resistance changes in asymmetric ionic conditions compared to its value in symmetric ionic conditions.	☐	☐
Lipid resistivity is a function of the thickeness of the bilayer.	☐	☐

Ionic channels

	T	F
Ionic channels formed by gramicidin molecules behave as ohmic conductors.	☐	☐
The total conductance is obtained by multiplying the total number of channels by the unitary conductance and by the open propability.	☐	☐
Unitary currents are zero at a 0 mV holding potential in asymmetric ionic conditions.	☐	☐
